# Long-Term Factors Associated With Falls and Fractures Poststroke

**DOI:** 10.3389/fneur.2018.00210

**Published:** 2018-04-03

**Authors:** Emma J. Foster, Raphae S. Barlas, Joao H. Bettencourt-Silva, Allan B. Clark, Anthony K. Metcalf, Kristian M. Bowles, John F. Potter, Phyo K. Myint

**Affiliations:** ^1^Ageing Clinical and Experimental Research (ACER) Team, School of Medicine, Medical Sciences & Nutrition, Institute of Applied Health Sciences, University of Aberdeen, Aberdeen, United Kingdom; ^2^Norfolk and Norwich University Hospital, Norwich, United Kingdom; ^3^Norwich Cardiovascular Research Group, Norwich Medical School, University of East Anglia, Norwich Research Park, Norwich, United Kingdom

**Keywords:** falls, fracture, stroke, risk, factor

## Abstract

**Background:**

Risk factors for poststroke falls and fractures remain poorly understood. This study aimed to evaluate which factors increased risk of these events after stroke.

**Methods:**

Data from 7,267 hospitalized stroke patients were acquired from the Norfolk and Norwich University Hospital Stroke Register from 2003–2015. The impacts of multiple patient level and stroke characteristics and comorbidities on post-discharge falls and fractures were assessed. Univariate and multivariable models were constructed, adjusting for multiple confounders, using binary logistic regression for short-term analysis (up to 1-year post-discharge) and Cox-proportional hazard models for longer term analysis (1–3, 3–5, and 0–10 years follow-up).

**Results:**

The mean age (SD) was 76.3 ± 12.1 years at baseline. 1,138 (15.7%) participants had an incident fall; and 666 (9.2%) an incident fracture during the 10-year follow-up (total person years = 64,447.99 for falls and 67,726.70 for fractures). Half of the sample population were females (50.6%) and the majority had an ischemic stroke (89.8%). After adjusting for confounders: age, sex, previous history of falls, and atrial fibrillation were associated with an increased risk of both falls and fractures during follow-up. Furthermore, chronic kidney disease and hyperlipidemia were associated with an increased risk of falls, while previous stroke/transient ischemic attack increased fracture risk. Total anterior circulation stroke and a prestroke modified Rankin Scale score of 3–5 were associated with decreased risk of both events, with hypertension and cancer decreasing risk of falls only.

**Conclusion:**

We identified demographic, stroke-related, and comorbid factors associated with poststroke falls and fracture incidence. Further studies are required to examine and establish the relationship between reversible factors and further explore the role of preventative measures to prevent poststroke falls and fractures.

## Introduction

Functional decline subsequent to acute stroke has been associated with an increased risk of falls (1–5) and reduction in bone mineral density after stroke has been well documented as a risk factor for fractures (6–8). Falls and fractures are major causes of functional decline, poor quality of life, frailty, dependency, and mortality. It is, therefore, important to better understand the risk factors for poststroke falls and fractures to develop intervention strategies, which can have substantial health benefit.

However, previous studies assessing such risk factors have several limitations. First, risk factor studies for falls have only been conducted over the short-term—such as the 1 and 6 month analyses conducted by Kerse et al. (9). Second, limited comorbidity adjustment has been an important limitation in previous studies; Lin et al. in their assessment of hip fracture after stroke, adjusted for relatively few variables (10)—including only six comorbidities (hypertension, diabetes, parkinsonism, epilepsy, dementia, and depression), age, sex, stroke type, and five categories of medication, therefore, omitting potentially important factors and resulting in increased risk of confounding. Third, small sample sizes resulted in limited statistical power, for example, Mackintosh et al. (11), including only 55 patients in their final analyses, as a more extreme example. Finally, previous studies have focused on investigating fall risk in relation to physical assessments, such as the Berg-Balance Scale or Timed Up and Go tests, which require additional time and resources to be applied in the clinical setting and are also perhaps irrelevant to the majority of stroke patients with significant disabilities (3, 11–16).

The current study, therefore, aimed to evaluate the association of a variety of potential risk factors, including demographic variables, stroke-related factors, and comorbidities, and falls and fracture risk over the long-term using a large, prospectively collected dataset with consecutive stroke admissions controlling for multiple confounders. An association between these factors and an increased risk of falls and fractures may facilitate the provision of prognostic information on the important long-term sequelae of stroke as well as aid the implementation of targeted preventive strategies to improve acute stroke outcomes.

## Materials and Methods

The study population was drawn from the Norfolk and Norwich University Hospital (NNUH) Stroke Register and included all consecutive stroke admissions from January 2003 to April 2015. NNUH is a regional tertiary hospital with a catchment population of approximately 750,000 people in the Norfolk (East Anglia) Region, UK. Record linkage enabled the ascertainment of comorbidity and mortality data, over a long term follow-up. The register received research database ethical approval from the Newcastle and Tyneside National Health Service and Research Ethics Committee (12/NE/0170), with the study protocol being approved by the Steering Committee of the Register.

The data collection methods have previously been described (17). Supervised by stroke specialists, the data entry team transferred paper and electronic records onto the stroke register database prospectively. Patients were included if a diagnosis of acute stroke had been confirmed by a combination of clinical examination, medical history, and neuroimaging. The prestroke modified Rankin Scale (mRS) score was confirmed from medical records. Follow-up for mortality data was obtained by electronic record linkage with the Office of National Statistics data.

The predictor variables of interest in this study were; age, sex, stroke type (ischemic or hemorrhagic), Oxfordshire Community Stroke Project (OCSP) classification, prestroke mRS score, previous fall, fracture, stroke or transient ischemic attack (TIA), congestive heart failure, coronary heart disease or myocardial infarction, atrial fibrillation, diabetes, hypertension, peripheral vascular disease, chronic kidney disease, chronic obstructive pulmonary disease, dementia, hyperlipidemia, and cancer. The outcomes of interest were first incident fall and fracture after hospital discharge from the index stroke episode occurring over the follow-up period. International Classification of Diseases (ICD-10) codes included for falls were W00 to W19 and the codes included for fractures were S82, S72, S62, S52, S42, S32, S22, S12, S02, T14.2, and T10.

### Statistical Analysis

Statistical analysis was performed using SPSS Statistics (version 24, IBM Corp., Armonk, New York, NY, USA). Descriptive statistics were calculated separately for those who had a fall or a fracture after stroke with categorical variables presented as the number and given percentage and continuous variables expressed as the mean ± SD. The association between falls and fractures and the risk factors under evaluation were assessed using the chi-squared and *t*-tests for categorical and continuous data, respectively. Univariate and multivariable models were constructed to calculate unadjusted and adjusted odds ratios from 0 to 365 days (0–1 year, short term) using binary logistic regression, and hazard ratios from 366 to 1,095 days (1–3 years, medium term), 1,096–1,825 days (3–5 years, medium-long term), and the full follow-up period from 0 to 3,650 days (0–10 years, long term) using Cox-proportional hazards. Univariate analyses were conducted by individually examining the association of each factor in a regression model alone on the outcomes of post-discharge falls and fractures; whereas for multivariable analyses, all variables were input together at the same time, therefore, examining their combined effect on the outcomes.

## Results

A total of 11,729 stroke cases were recorded between January 2003 and April 2015. Participants were excluded if they did not meet the eligibility criteria for the study, leaving a total of 7,267 people (Figure S1 in Supplementary Material). The mean age (SD) of the study population at baseline was 76.3 ± 12.1 years and 50.6% (3,678) were females. Ischemic stroke occurred in 89.8% of cases, while the most common OCSP stroke classification was partial anterior circulation stroke (PACS) (37.8%). The majority of patients were completely independent before their stroke with a prestroke mRS score of 0 (67.5%). 1,138 (15.7%) of patients suffered incident falls and 666 (9.2%) had an incident fracture post-discharge. Approximately two-thirds of both falls and fractures occurred in women (60.7 and 65.0%, respectively).

Table 1 shows the baseline characteristics for patients who had a post-discharge fall or fracture after stroke compared to those who did not. Both falls and fractures were associated with increasing age (*P* < 0.001); 45.1% of falls and 45.8% of fractures occurred in the 81–89 years old age group. Regarding OCSP classification, falls and fractures were significantly associated with less severe stroke, as most events occurred in the lacunar stroke (LACS) or PACS sub-types (30.8 and 41.0% for falls and 30.5 and 40.2% for fractures, respectively). Both events were associated with a low prestroke mRS score, with over 60% of falls and fractures being found in those with a score of 0. A history of falls and fractures were both associated with increased incidence of post-discharge falls and fractures (*P* < 0.001). In addition, previous stroke/TIA and hypertension were associated with fractures and atrial fibrillation was associated with both falls and fractures.

**Table 1:** Characteristics of patients with and without post-discharge falls and fractures.

	Fall		*P*	Fracture		*P*
	No (%)	Yes (%)		No (%)	Yes (%)	
Age^a^	75.48 (±12.47)	80.44 (±9.20)	<0.001	75.88 (±12.30)	80.07 (±9.72)	<0.001
**Age group**						
<65	1,111 (18.1)	71 (6.2)	<0.001	1,127 (17.1)	55 (8.3)	<0.001
65–70	718 (11.7)	78 (6.9)		755 (11.4)	41 (6.2)	
71–80	1,831 (29.9)	331 (29.1)		1,978 (30.0)	184 (27.6)	
81–89	1,893 (30.9)	513 (45.1)		2,101 (31.8)	305 (45.8)	
90+	576 (9.4)	145 (12.7)		640 (9.7)	81 (12.2)	

**Sex**						
Women	2,987 (48.7)	691 (60.7)	<0.001	3,245 (49.2)	433 (65.0)	<0.001
Men	3,142 (51.3)	447 (39.3)		3,356 (50.8)	233 (35.0)	

**Type of stroke**						
Infarct	5,495 (89.7)	1,028 (90.3)	0.522	5,921 (89.7)	602 (90.4)	0.621
Hemorrhage	634 (10.3)	110 (9.7)		680 (10.3)	64 (9.6)	

**Oxfordshire community stroke project classification**						
Lacunar stroke	1,665 (27.2)	350 (30.8)	<0.001	1,812 (27.5)	203 (30.5)	0.007
Partial anterior circulation stroke	2,282 (37.2)	467 (41.0)		2,481 (37.6)	268 (40.2)	
Posterior circulation stroke	1,127 (18.4)	182 (16.0)		1,199 (18.2)	110 (16.5)	
Total anterior circulation stroke	840 (13.7)	102 (9.0)		883 (13.4)	59 (8.9)	
Undetermined	215 (3.5)	37 (3.3)		226 (3.4)	26 (3.9)	

**Prestroke modified Rankin score**						
0	4,200 (68.5)	708 (62.2)	<0.001	4,488 (68.0)	420 (63.1)	0.027
1–2	1,138 (18.6)	263 (23.1)		1,250 (18.9)	151 (22.7)	
3–5	791 (12.9)	167 (14.7)		863 (13.1)	95 (14.3)	
Previous fall	656 (10.7)	207 (18.2)	<0.001	714 (10.8)	149 (22.4)	<0.001
Previous fracture	470 (7.7)	142 (12.5)	<0.001	509 (7.7)	103 (15.5)	<0.001

**Comorbidities**						
Previous stroke or transient ischemic attack	637 (10.4)	137 (12.0)	0.110	679 (10.3)	95 (14.3)	0.002
Congestive heart failure	408 (6.7)	74 (6.5)	0.899	429 (6.5)	53 (8.0)	0.174
Coronary heart disease or myocardial infarction	953 (15.5)	193 (17.0)	0.248	1,026 (15.5)	120 (18.0)	0.106
Atrial fibrillation	737 (12.0)	169 (14.9)	0.009	798 (12.1)	108 (16.2)	0.003
Diabetes	564 (9.2)	109 (9.6)	0.729	607 (9.2)	66 (9.9)	0.592
Hypertension	1,747 (28.5)	351 (30.8)	0.118	1,879 (28.5)	219 (32.9)	0.019
Peripheral vascular disease	117 (1.9)	22 (1.9)	>0.999	123 (1.9)	16 (2.4)	0.412
Chronic kidney disease	160 (2.6)	36 (3.2)	0.338	175 (2.7)	21 (3.2)	0.524
Chronic obstructive pulmonary disease	239 (3.9)	42 (3.7)	0.801	256 (3.9)	25 (3.8)	0.957
Dementia	149 (2.4)	34 (3.0)	0.318	165 (2.5)	18 (2.7)	0.850
Hyperlipidemia	240 (3.9)	55 (4.8)	0.174	265 (4.0)	30 (4.5)	0.612
Cancer	655 (10.7)	103 (9.1)	0.108	692 (10.5)	66 (9.9)	0.693

*^a^Presented as mean (±SD)*.

Table 2 shows the results of the univariate analysis for risk factors for poststroke falls after discharge over the prespecified time periods. The total many factors were associated with post-discharge fall risk in these unadjusted analyses, with the risk profile changing over follow-up. Table 3 displays the results of the fully adjusted multivariable analysis for risk factors for poststroke falls after discharge. Increasing age was associated with an increased risk of falls at all time periods. Female sex and a history of falls were associated with an increased risk of falls at three of the time periods (0–1, 1–3, and 0–10 years). Total anterior circulation strokes (TACS) were associated with reduced risk of falls from 3 to 5 years and over the whole follow-up. A prestroke mRS score of 3–5 (indicating moderate–severe disability) was also associated with reduced risk of falls over the whole follow-up period of 0–10 years. Chronic kidney disease was associated with an increased falls risk at 0–1 year and atrial fibrillation at 1–3 years only. Hyperlipidemia was associated with increased risk of falls over the whole follow-up period of 0–10 years—HR 1.35 and 95% CI (1.01–1.80), *P* = 0.042—and borderline increased risk at 1–3 years [HR 1.59, 95% CI (1.00–2.55), *P* = 0.052]. Conversely, a history of hypertension and cancer showed reduced fall risk at 1–3 years and over the whole follow-up, respectively.

**Table 2:** Univariate analysis of risk factors for poststroke falls over time after discharge.

	0–365 days	366–1,095 days	1,096–1,825 days	0–3,650 days
	OR (95% CI)	HR (95% CI)	HR (95% CI)	HR (95% CI)
Age	1.07 (1.05–1.08)***	1.06 (1.05–1.07)***	1.05 (1.03–1.06)***	1.04 (1.03–1.05)***
**Sex**				
Male	1.00	1.00	1.00	1.00
Female	1.80 (1.44–2.27)***	1.82 (1.47–2.24)***	1.48 (1.13–1.94)**	1.58 (1.40–1.77)***

**Type of stroke**				
Infarct	1.00	1.00	1.00	1.00
Hemorrhage	1.02 (0.71–1.46)	0.76 (0.53–1.11)	0.81 (0.51–1.30)	0.93 (0.77–1.13)

**Oxfordshire community stroke project classification**				
Lacunar stroke	1.00	1.00	1.00	1.00
Partial anterior circulation stroke	1.32 (1.00–1.74)	1.04 (0.82–1.32)	0.84 (0.61–1.15)	0.99 (0.86–1.13)
Posterior circulation stroke	1.17 (0.83–1.64)	0.63 (0.45–0.88)**	0.80 (0.55–1.17)	0.79 (0.66–0.95)*
Total anterior circulation stroke	1.06 (0.72–1.56)	0.77 (0.53–1.11)	0.51 (0.29–0.88)*	0.61 (0.49–0.76)***
Undetermined	0.99 (0.51–1.93)	1.16 (0.69–1.95)	0.75 (0.33–1.72)	0.84 (0.60–1.18)

**Prestroke modified Rankin score**				
0	1.00	1.00	1.00	1.00
1–2	1.88 (1.45–2.44)***	1.82 (1.42–2.32)***	1.75 (1.28–2.40)***	1.36 (1.18–1.56)***
3–5	2.13 (1.60–2.84)***	2.47 (1.89–3.24)***	1.11 (0.66–1.85)	1.27 (1.07–1.51)**

Previous fall	2.54 (1.96–3.30)***	2.72 (2.13–3.47)***	1.65 (1.11–2.48)*	1.80 (1.55–2.09)***
Previous fracture	2.24 (1.66–3.03)***	2.25 (1.68–3.00)***	1.94 (1.27–2.97)**	1.67 (1.40–1.99)***
Previous stroke or transient ischemic attack	1.58 (1.16–2.14)**	1.23 (0.90–1.69)	1.35 (0.89–2.05)	1.18 (0.99–1.41)
Congestive heart failure	1.46 (1.00–2.14)	1.61 (1.09–2.36)*	1.39 (0.78–2.50)	1.00 (0.79–1.26)
Coronary heart disease or myocardial infarction	1.43 (1.09–1.88)**	1.28 (0.98–1.67)	1.39 (0.98–1.97)	1.11 (0.96–1.30)
Atrial fibrillation	1.63 (1.23–2.17)**	1.83 (1.40–2.39)***	1.17 (0.76–1.80)	1.27 (1.08–1.49)**
Diabetes	1.47 (1.06–2.04)*	1.19 (0.85–1.67)	1.15 (0.72–1.82)	1.06 (0.87–1.29)
Hypertension	1.53 (1.22–1.92)***	1.19 (0.96–1.48)	1.33 (1.00–1.77)	1.12 (0.99–1.27)
Peripheral vascular disease	1.10 (0.51–2.36)	1.48 (0.77–2.87)	0.96 (0.31–3.01)	1.02 (0.67–1.56)
Chronic kidney disease	2.57 (1.61–4.10)***	1.34 (0.72–2.51)	1.04 (0.39–2.79)	1.25 (0.89–1.74)
Chronic obstructive pulmonary disease	1.71 (1.08–2.71)*	1.06 (0.61–1.84)	1.25 (0.62–2.53)	0.97 (0.72–1.33)
Dementia	2.47 (1.52–4.03)***	2.07 (1.19–3.60)*	0.59 (0.15–2.37)	1.27 (0.90–1.79)
Hyperlipidemia	1.45 (0.90–2.33)	1.45 (0.93–2.25)	1.46 (0.82–2.62)	1.24 (0.95–1.63)
Cancer	1.00 (0.70–1.43)	0.96 (0.67–1.38)	1.01 (0.62–1.63)	0.85 (0.69–1.04)

*P < 0.05, **P < 0.01, ***P < 0.001.

**Table 3:** Multivariable analysis of risk factors for poststroke falls over time after discharge.

	0–365 days		366–1,095 days		1,096–1,825 days		0–3,650 days	
	OR (95% CI)	*P*	HR (95% CI)	*P*	HR (95% CI)	*P*	HR (95% CI)	*P*
Age	1.06 (1.04–1.07)	<0.001	1.05 (1.04–1.06)	<0.001	1.05 (1.03–1.06)	<0.001	1.04 (1.03–1.04)	<0.001
**Sex**								
Male	1.00	0.031	1.00	0.024	1.00	0.275	1.00	<0.001
Female	1.31 (1.03–1.67)		1.29 (1.04–1.62)		1.17 (0.88–1.56)		1.29 (1.14–1.47)	

**Type of stroke**								
Infarct	1.00	0.254	1.00	0.673	1.00	0.762	1.00	0.469
Hemorrhage	1.24 (0.86–1.81)		0.92 (0.63–1.35)		0.93 (0.57–1.51)		1.08 (0.88–1.32)	

**Oxfordshire community stroke project classification**								
Lacunar stroke	1.00		1.00		1.00		1.00	
Partial anterior circulation stroke	1.25 (0.94–1.66)	0.127	0.99 (0.78–1.26)	0.962	0.80 (0.58–1.10)	0.168	0.94 (0.82–1.08)	0.393
Posterior circulation stroke	1.35 (0.95–1.92)	0.090	0.75 (0.54–1.06)	0.101	0.90 (0.61–1.33)	0.603	0.87 (0.72–1.04)	0.125
Total anterior circulation stroke	1.02 (0.69–1.51)	0.935	0.74 (0.51–1.08)	0.122	0.48 (0.27–0.84)	0.009	0.58 (0.46–0.72)	<0.001
Undetermined	1.05 (0.53–2.10)	0.886	1.29 (0.76–2.20)	0.348	0.91 (0.39–2.11)	0.817	0.90 (0.64–1.28)	0.560

**Prestroke modified Rankin score**								
0	1.00		1.00		1.00		1.00	
1–2	1.25 (0.95–1.65)	0.112	1.27 (0.98–1.64)	0.070	1.32 (0.95–1.83)	0.103	1.06 (0.91–1.22)	0.471
3–5	1.05 (0.76–1.46)	0.767	1.31 (0.96–1.78)	0.087	0.74 (0.43–1.29)	0.287	0.80 (0.67–0.97)	0.023
Previous fall	1.49 (1.03–2.17)	0.035	1.65 (1.17–2.33)	0.004	0.94 (0.52–1.69)	0.837	1.38 (1.11–1.71)	0.003
Previous fracture	1.03 (0.68–1.57)	0.882	1.04 (0.71–1.54)	0.830	1.60 (0.88–2.92)	0.127	1.05 (0.82–1.33)	0.702
Previous stroke or transient ischemic attack	1.26 (0.91–1.75)	0.159	0.96 (0.69–1.34)	0.817	1.15 (0.76–1.79)	0.521	1.08 (0.90–1.30)	0.413
Congestive heart failure	0.81 (0.52–1.25)	0.334	1.07 (0.70–1.65)	0.745	1.11 (0.60–2.10)	0.744	0.77 (0.60–1.00)	0.054
Coronary heart disease or myocardial infarction	1.04 (0.76–1.42)	0.816	0.99 (0.73–1.35)	0.950	1.18 (0.79–1.77)	0.417	1.02 (0.85–1.22)	0.839
Atrial fibrillation	1.07 (0.77–1.49)	0.677	1.46 (1.08–1.98)	0.015	0.87 (0.54–1.40)	0.566	1.14 (0.95–1.37)	0.154
Diabetes	1.25 (0.88–1.79)	0.220	1.15 (0.80–1.65)	0.449	1.04 (0.63–1.69)	0.892	1.06 (0.86–1.30)	0.614
Hypertension	0.96 (0.73–1.26)	0.773	0.73 (0.56–0.94)	0.016	0.98 (0.70–1.38)	0.910	0.88 (0.76–1.02)	0.100
Peripheral vascular disease	0.74 (0.33–1.64)	0.454	1.27 (0.64–2.50)	0.492	0.86 (0.27–2.75)	0.804	0.96 (0.62–1.48)	0.849
Chronic kidney disease	1.98 (1.19–3.30)	0.009	0.97 (0.50–1.87)	0.917	0.82 (0.29–2.28)	0.698	1.23 (0.86–1.74)	0.255
Chronic obstructive pulmonary disease	1.40 (0.86–2.27)	0.171	0.84 (0.47–1.47)	0.536	1.04 (0.50–2.15)	0.917	0.92 (0.67–1.26)	0.613
Dementia	1.15 (0.68–1.96)	0.601	0.80 (0.45–1.43)	0.449	0.37 (0.09–1.52)	0.167	0.86 (0.60–1.23)	0.406
Hyperlipidemia	1.37 (0.81–2.30)	0.238	1.59 (1.00–2.55)	0.052	1.39 (0.74–2.60)	0.309	1.35 (1.01–1.80)	0.042
Cancer	0.79 (0.55–1.15)	0.216	0.82 (0.57–1.19)	0.296	0.80 (0.49–1.31)	0.373	0.76 (0.61–0.93)	0.008

Table 4 displays the results of the univariate analysis for risk factors for poststroke fractures after discharge over the prespecified time periods. Once again, many factors were associated with post-discharge fracture risk in these unadjusted analyses, with the risk profile changing over follow-up. Table 5 shows the results of the fully adjusted multivariable analyses for poststroke fracture risk factors after discharge. Increasing age was associated with an increased risk of fractures at all four time periods. Female sex and a history of falls were also associated with an increased risk of fractures at 0–1, 1–3, and 0–10 years. Atrial fibrillation was also associated with increased risk of fracture from 1 to 3 years after discharge only [HR 1.62, 95% CI (1.09–2.40), *P* = 0.017] and previous stroke/TIA were associated with increased risk over the whole follow-up [HR 1.27, 95% CI (1.01–1.60), *P* = 0.041] (also just failing to reach statistical significance at 0–1 years after discharge). Once again, TACS were associated with decreased risk of fracture from 1 to 3 years and over the whole follow-up period (again with borderline significance during the 3–5-year time period). A prestroke mRS score of 3–5 was also associated with reduced risk of fracture over the whole follow-up of 0–10 years.

**Table 4:** Univariate analysis of risk factors for poststroke fractures over time after discharge.

	0–365 days	366–1,095 days	1,096–1,825 days	0–3,650 days
	OR (95% CI)	HR (95% CI)	HR (95% CI)	HR (95% CI)
Age	1.07 (1.05–1.09)***	1.04 (1.03–1.06)***	1.05 (1.03–1.06)***	1.03 (1.03–1.04)***
**Sex**				
Male	1.00	1.00	1.00	1.00
Female	2.30 (1.73–3.05)***	1.89 (1.42–2.51)***	1.75 (1.20–2.55)**	1.88 (1.60–2.20)***

**Type of stroke**								
Infarct	1.00	1.00	1.00	1.00
Hemorrhage	1.05 (0.69–1.60)	0.75 (0.45–1.25)	0.71 (0.36–1.40)	0.93 (0.72–1.20)

**Oxfordshire community stroke project classification**				
Lacunar stroke	1.00	1.00	1.00	1.00
Partial anterior circulation stroke	1.21 (0.87–1.69)	0.86 (0.63–1.18)	0.95 (0.62–1.48)	0.97 (0.81–1.16)
Posterior circulation stroke	0.99 (0.66–1.50)	0.62 (0.40–0.95)*	0.93 (0.55–1.56)	0.83 (0.66–1.04)
Total anterior circulation stroke	1.09 (0.70–1.70)	0.56 (0.33–0.97)*	0.49 (0.22–1.09)	0.62 (0.46–0.82)*
Undetermined	1.37 (0.69–2.71)	1.22 (0.63–2.36)	1.32 (0.52–3.35)	1.04 (0.69–1.56)

**Prestroke modified Rankin score**				
0	1.00	1.00	1.00	1.00
1–2	2.13 (1.57–2.89)***	1.56 (1.12–2.18)**	1.45 (0.93–2.27)	1.29 (1.07–1.56)**
3–5	2.34 (1.67–3.28)***	1.66 (1.11–2.48)*	1.31 (0.70–2.45)	1.19 (0.96–1.49)
Previous fall	4.15 (3.13–5.49)***	3.01 (2.18–4.15)***	1.67 (0.97–2.87)	2.32 (1.93–2.78)***
Previous fracture	3.76 (2.76–5.13)***	2.33 (1.58–3.43)***	2.10 (1.20–3.67)**	2.15 (1.74–2.65)***
Previous stroke or transient ischemic attack	1.88 (1.34–2.65)***	1.37 (0.91–2.07)	1.66 (0.98–2.82)	1.43 (1.15–1.78)**
Congestive heart failure	1.80 (1.19–2.74)**	1.69 (1.01–2.81)*	1.96 (0.99–3.86)	1.25 (0.94–1.65)
Coronary heart disease or myocardial infarction	1.40 (1.01–1.93)*	1.43 (1.01–2.02)*	1.67 (1.07–2.62)*	1.19 (0.98–1.45)
Atrial fibrillation	1.76 (1.26–2.44)**	2.04 (1.44–2.89)***	1.10 (0.61–2.00)	1.39 (1.13–1.71)**
Diabetes	1.29 (0.86–1.94)	1.50 (0.99–2.28)	0.61 (0.27–1.38)	1.09 (0.84–1.40)
Hypertension	1.51 (1.15–1.97)**	1.44 (1.08–1.92)*	1.46 (0.99–2.14)	1.23 (1.04–1.44)*
Peripheral vascular disease	2.12 (1.07–4.23)*	1.55 (0.64–3.76)	0.59 (0.08–4.25)	1.30 (0.79–2.14)
Chronic kidney disease	2.40 (1.37–4.19)**	1.18 (0.49–2.87)	0.45 (0.06–3.24)	1.21 (0.78–1.87)
Chronic obstructive pulmonary disease	1.74 (1.02–2.98)*	0.74 (0.31–1.80)	1.14 (0.42–3.08)	0.98 (0.66–1.46)
Dementia	1.97 (1.05–3.67)*	1.35 (0.56–3.27)	1.07 (0.26–4.33)	1.10 (0.69–1.76)
Hyperlipidemia	1.29 (0.71–2.33)	1.48 (0.83–2.65)	1.08 (0.44–2.66)	1.13 (0.78–1.63)
Cancer	0.89 (0.57–1.39)	1.24 (0.80–1.93)	1.29 (0.71–2.35)	0.94 (0.73–1.21)

*P < 0.05, **P < 0.01, ***P < 0.001.

**Table 5:** Multivariable analysis of risk factors for poststroke fractures over time after discharge.

	0–365 days		366–1,095 days		1,096–1,825 days		0–3,650 days	
	OR (95% CI)	*P*	HR (95% CI)	*P*	HR (95% CI)	*P*	HR (95% CI)	*P*
Age	1.05 (1.03–1.07)	<0.001	1.03 (1.02–1.05)	<0.001	1.04 (1.02–1.06)	<0.001	1.03 (1.02–1.03)	<0.001
**Sex**								
Male	1.00	0.003	1.00	0.007	1.00	0.098	1.00	<0.001
Female	1.58 (1.16–2.13)		1.52 (1.12–2.05)		1.40 (0.94–2.08)		1.59 (1.34–1.88)	

**Type of stroke**								
Infarct	1.00	0.251	1.00	0.630	1.00	0.425	1.00	0.768
Hemorrhage	1.30 (0.83–2.02)		0.88 (0.52–1.49)		0.75 (0.37–1.52)		1.04 (0.80–1.36)	

**Oxfordshire community stroke project classification**								
Lacunar stroke	1.00		1.00		1.00		1.00	
Partial anterior circulation stroke	1.14 (0.82–1.60)	0.439	0.82 (0.60–1.13)	0.230	0.91 (0.59–1.41)	0.668	0.93 (0.77–1.11)	0.422
Posterior circulation stroke	1.17 (0.77–1.80)	0.465	0.72 (0.47–1.12)	0.144	1.07 (0.63–1.81)	0.805	0.92 (0.72–1.16)	0.461
Total anterior circulation stroke	1.05 (0.66–1.65)	0.848	0.54 (0.32–0.94)	0.028	0.45 (0.20–1.02)	0.056	0.58 (0.43–0.78)	<0.001
Undetermined	1.45 (0.71–2.96)	0.308	1.39 (0.70–2.75)	0.342	1.69 (0.65–4.40)	0.285	1.11 (0.73–1.69)	0.628

**Prestroke modified Rankin score**								
0	1.00		1.00		1.00		1.00	
1–2	1.31 (0.95–1.82)	0.102	1.08 (0.76–1.53)	0.676	1.07 (0.67–1.71)	0.787	0.96 (0.79–1.17)	0.705
3–5	1.01 (0.69–1.49)	0.963	0.87 (0.55–1.35)	0.525	0.82 (0.42–1.63)	0.577	0.71 (0.55–0.90)	0.006
Previous fall	2.20 (1.45–3.33)	<0.001	2.32 (1.47–3.64)	<0.001	0.81 (0.37–1.79)	0.604	1.77 (1.36–2.30)	<0.001
Previous fracture	1.33 (0.86–2.08)	0.202	0.93 (0.55–1.56)	0.781	1.84 (0.83–4.07)	0.132	1.14 (0.85–1.53)	0.376
Previous stroke or transient ischemic attack	1.44 (0.99–2.09)	0.054	1.02 (0.66–1.56)	0.945	1.43 (0.82–2.50)	0.206	1.27 (1.01–1.60)	0.041
Congestive heart failure	1.02 (0.63–1.66)	0.936	1.10 (0.63–1.93)	0.739	1.67 (0.79–3.55)	0.180	0.96 (0.70–1.32)	0.806
Coronary heart disease or myocardial infarction	0.96 (0.66–1.40)	0.825	1.05 (0.70–1.57)	0.814	1.51 (0.90–2.53)	0.119	1.05 (0.83–1.31)	0.700
Atrial fibrillation	1.11 (0.76–1.63)	0.595	1.62 (1.09–2.40)	0.017	0.70 (0.36–1.35)	0.290	1.16 (0.91–1.46)	0.227
Diabetes	1.10 (0.70–1.71)	0.681	1.38 (0.88–2.16)	0.159	0.52 (0.22–1.22)	0.131	1.06 (0.81–1.38)	0.694
Hypertension	0.86 (0.62–1.19)	0.370	0.89 (0.63–1.26)	0.513	1.12 (0.72–1.77)	0.612	0.92 (0.76–1.12)	0.400
Peripheral vascular disease	1.62 (0.77–3.39)	0.205	1.19 (0.48–2.98)	0.708	0.59 (0.08–4.29)	0.601	1.22 (0.73–2.03)	0.444
Chronic kidney disease	1.70 (0.92–3.16)	0.092	0.76 (0.30–1.92)	0.560	0.35 (0.05–2.58)	0.302	1.08 (0.68–1.70)	0.757
Chronic obstructive pulmonary disease	1.32 (0.75–2.34)	0.341	0.57 (0.23–1.41)	0.224	0.92 (0.33–2.54)	0.867	0.87 (0.58–1.31)	0.511
Dementia	0.73 (0.37–1.42)	0.355	0.59 (0.24–1.49)	0.267	0.70 (0.16–3.01)	0.635	0.70 (0.43–1.14)	0.152
Hyperlipidemia	1.21 (0.63–2.30)	0.566	1.35 (0.72–2.52)	0.347	1.00 (0.39–2.57)	0.998	1.11 (0.76–1.64)	0.590
Cancer	0.73 (0.46–1.17)	0.190	1.05 (0.66–1.65)	0.842	1.05 (0.57–1.95)	0.870	0.86 (0.66–1.11)	0.243

Table 6 indicates the number of events over the total number of person years follow-up within each time period. The total number of patients in each analysis from 0 to 1 to 3–5 years decreased due to the exclusion of patients who had either suffered an event or died in previous time periods to reduce the impact of previous events on later analyses and allowing clinicians to better understand factors associated with the falls and fracture risk based on patient’s survival.

**Table 6:** Displaying the number of fall and fracture events, over the total number of person years during each time period.

Time period	Falls/total number	Fractures/total number
0–365 days	336/7,090.10	234/7,130.22
366–1,095 days	379/13,451.20	207/13,853.89
1,096–1,825 days	216/12,870.42	116/13,526.41
0–3,650 days	1,138/64,447.99	666/67,726.70

## Discussion

Our study has found several factors to be associated with poststroke falls and fractures. The identified risk factors included; demographic features such as increasing age and female sex, and comorbidities such as a previous history of falls and atrial fibrillation, which were associated with increased risk of both events at various points during follow-up, additionally, chronic kidney disease and hyperlipidemia were associated with an increased risk of falls only, while previous stroke/TIA increased fracture risk. Conversely, increasing stroke severity (TACS) and premorbid disability depicted by prestroke mRS score 3–5 were associated with decreased risk of falls and fractures, while hypertension and cancer were associated with decreased risk of falls only. It can also be seen that there are shared risk factors with non-stroke populations.

Our findings, therefore, are in-keeping with previous studies where female sex, older age, and previous falls were found to be associated with an increased risk of falls and fractures after stroke (9, 10, 18, 19). Furthermore, the current study was able to replicate these findings while addressing some of the limitations inherent to some previous studies, such as short follow-up, small sample sizes, or limited comorbidity adjustment. Atrial fibrillation has also been shown to be associated with a history of falls in a study examining the relationships between falls history, atrial fibrillation, and mortality (20)—furthermore, it has also been linked to fracture risk in a large population-based study with robust comorbid adjustment (21) and also in the work by Kapral et al. investigating fracture risk after stroke (19). Atrial fibrillation is positively associated with fractures at 366–1,095 days poststroke (HR > 1), but shows a HR < 1 for the time interval 1,096–1,825 days poststroke. This apparent inconsistency may be due to a sample size issue in the later analyses and hence resulting in a type II error.

Our results showed that chronic kidney disease was a risk factor for falls within the first year after stroke discharge. This may be due to the loss of bone and muscle mass among other factors, such as polypharmacy, leading to frail patients with an increased risk of falls, as shown in non-stroke populations (22–24). After a stroke, there is also muscle wasting (25), which further contributes toward fall risk, with there being many shared risk factors between stroke and chronic kidney disease and an additional association with chronic kidney disease patients being at increased risk of stroke (26). As with people with atrial fibrillation, with reduced sample size in these comorbid groups, the direction of effect appeared to be protective beyond year 1 in people with chronic kidney disease. Further research should investigate if stroke patients with chronic kidney disease are indeed at higher risk in a larger sample and whether this is due to these combined effects leading to accelerated loss of muscle function. The current study also observed a history of previous stroke/TIA to be associated with increased fracture risk over follow-up, which may be explained by the reduced bone mass of stroke survivors (6–8, 27). Female sex has been previously linked to increased risk of being care dependent at 1 year after stroke as they are older and suffer from more geriatric comorbidities, female sex could, therefore, be seen as a marker of such factors not included in our study, which could further explain the associations shown (28). Finally, the current study found that hyperlipidemia was associated with increased risk of falls. This may be related to the use of statins, which have been previously associated with an increased risk of falls due to side-effects such as muscle pain and weakness (29); however, the current study did not collect data regarding statin use in patients and so this association remains uncertain.

Malignancy and associated therapies have been consistently associated with bone loss (30, 31). Furthermore, the side effects of cancer treatment, such as chemotherapy-induced peripheral neuropathy (32) or androgen deprivation therapy (e.g., possible loss of muscle mass) (33, 34) may lead to an increased risk of falls. However, the current study found an association between malignancy and a reduced risk of poststroke falls and no association with fractures, after adjusting for confounders. This may be due to the long follow-up of our study and the possible increased mortality of patients with a history of cancer during the short-term follow-up period. We also found that having a TACS or mRS score of 3–5 reduced fall and fracture risk. We acknowledge that the mRS score was not created to be applied to pre-stroke disability. However, we, and others, have shown the usefulness of mRS on prognosis (35, 36). For mRS 0, we define this as completely normal (without any disability). We have used the current classification to be in line with the literature (where mRS greater than or equal to 3 is regarded as an indicator of severe pre-stroke disability). These negative associations could, therefore, similarly be explained by the substantially reduced mobility of these patients or possibly increased mortality, who are, therefore, less at risk of an event. Hypertension was also negatively associated with falls risk from 1 to 3 years. A previous questionnaire-based study has also reported an observed reduced risk of falls in hypertensive women, and it has also been suggested that high blood pressure is associated with reduced physical and cognitive decline, which may account for this decreased risk (37, 38). However, the population in this study may not be representative as this concerns the oldest old. Hypertension is known to be a risk factor for cognitive decline (39) and, so, this relationship seen also remains unclear. We also did not account for patient use of antihypertensives, which may have altered the relationship observed. The mechanisms underlying this observed association, therefore, remain unclear.

Falls and fractures are associated with increased morbidity and mortality, and the risk factors identified in the current study can help predict patients at risk of poststroke falls and fractures, thereby facilitating the implementation of preventative strategies, such as exercises, medication reviews, or ensuring safe environments to improve outcomes (40).

Our study had some limitations. Bone mineral density decreases after stroke; however, we did not have these data and were, therefore, unable to adjust for this factor. We did not examine the specific type of fracture risk, which may have proved important; however, even with our large sample, specific fracture type analyses may have reduced the study power. Future studies are required to better understand the risk factors for specific fractures with higher diseases burden. Furthermore, there was no indication of comorbid disease severity, which could benefit the validity of our results. Selection bias may be a significant consideration in the interpretation of the analyses, due to the exclusion of a large proportion of the original cohort of patients. However, it should be noted that the majority of exclusions were due to inpatient deaths (and, therefore, inappropriate for our outcome of post-discharge events) or repeat admission of the same patient during follow-up (*N* = 3,395, 76.1% of all exclusions), as detailed (Figure S1 in Supplementary Material). Due to the methods of data collection and the ascertainment of falls and fractures based on ICD-10 codes, there could also have been unreported events or underestimation of their true prevalence among the cohort. Milder falls are likely to have been under-reported and thus may have different associated factors to those reported here, where events reported are likely to be more major. However, any underestimation of events would likely only have attenuated the associations seen. The cohort consisted of mainly white caucasians and whether these risk factors are applicable to other ethnic groups needs to be tested in an independent dataset with ethnic mix. Finally, as an observational study, we cannot exclude possibility of residual confounding.

Despite these limitations, our study had strengths in the robust model construction, and we were able to control for multiple confounders and comorbidities over a long duration follow-up of 10 years and our discrete period analysis also allows better understanding of the changing risk factor profile with time after stroke. In addition to this, we had a large sample size, which has allowed us to address some limitations in the previous literature.

## Conclusion

In summary, we have identified and confirmed risk factor findings of previous research in both stroke and non-stroke populations, such as age, female sex, and a history of falls, in a manner which takes into account discrete follow-up periods. The results from this study imply that these identified factors should be considered in stroke patients to identify those at increased risk of having a fall or fracture post-discharge. Since falls and fractures after stroke can have substantial adverse effects, identifying at-risk patients early on may allow us to target interventions toward these patients—such as medication reviews and ensuring safe mobility—and thus may bring substantial benefit to patients with stroke. Further studies are required to examine and establish the relationship between reversible factors and further explore the role of preventative measures to prevent poststroke falls and fractures.

## Data Availability

Data are available on request from the Norfolk and Norwich University Stroke and TIA Register Steering Committee.

## Author Contributions

PM, EF, and RB contributed to conception and design of the study. The dataset used in this study is maintained by our colleagues at Norfolk and Norwich (JB-S, AC, AM, KB, and JP). Statistical analyses were performed by EF and supervised by RB. EF wrote the first draft of the manuscript, which was redrafted by EF, RB, PM, and JP. All authors contributed to, read, and approved the final draft of the manuscript for submission. PM is the guarantor.

## Conflict of Interest Statement

The authors declare that the research was conducted in the absence of any commercial or financial relationships that could be construed as a potential conflict of interest.

## References

[1] Forster A Young J. Incidence and consequences of falls due to stroke: a systematic inquiry. BMJ (1995) 311:83. 10.1136/bmj.311.6997.83 7613406 PMC2550147

[2] Jørgensen L Engstad T Jacobsen B Higher incidence of falls in long-term stroke survivors than in population controls. Stroke (2002) 33:542–7. 10.1161/hs0202.102375 11823667

[3] Ashburn A Hyndman D Pickering R Yardley L Harris S. Predicting people with stroke at risk of falls. Age Ageing (2008) 37(3):270–6. 10.1093/ageing/afn066 18456791

[4] Schmid AA Yaggi HK Burrus N McClain V Austin C Ferguson J Circumstances and consequences of falls among people with chronic stroke. J Rehabil Res Dev (2013) 50(9):1277–86. 10.1682/JRRD.2012.11.0215 24458967

[5] Chang VC Do MT Risk factors for falls among seniors: implications of gender. Am J Epidemiol (2015) 181:521–31. 10.1093/aje/kwu268 25700887

[6] Moayyeri A Alrawi YA Myint PK. The complex mutual connection between stroke and bone health. Arch Biochem Biophys (2010) 503(1):153–9. 10.1016/j.abb.2010.06.023 20599661

[7] Myint PK Clark AB Kwok CS Loke YK Yeong JK Luben RN Bone mineral density and incidence of stroke: European prospective investigation into cancer-Norfolk population-based study, systematic review, and meta-analysis. Stroke (2014) 45(2):373–82. 10.1161/STROKEAHA.113.002999 24399373

[8] Huo K Hashim S Yong K Su H Qu Q. Impact and risk factors of post-stroke bone fracture. World J Exp Med (2016) 6(1):1–8. 10.5493/wjem.v6.i1.1 26929915 PMC4759351

[9] Kerse N Parag V Feigin V McNaughton H Hackett M Bennett D Falls after stroke: results from the Auckland Regional Community Stroke (ARCOS) study, 2002 to 2003. Stroke (2008) 39(6):1890–3. 10.1161/STROKEAHA.107.509885 18483413

[10] Lin HL Lin HC Tseng YF Liao HH Worly JA Pan CY Hip fracture after first-ever stroke: a population-based study. Acta Neurol Scand (2015) 131(3):158–63. 10.1111/ane.12301 25263230

[11] Mackintosh SF Hill KD Dodd KJ Goldie PA Culham EG. Balance score and a history of falls in hospital predict recurrent falls in the 6 months following stroke rehabilitation. Arch Phys Med Rehabil (2006) 87(12):1583–9. 10.1016/j.apmr.2006.09.004 17141637

[12] Persson CU Hansson PO Sunnerhagen KS. Clinical tests performed in acute stroke identify the risk of falling during the first year: posturalstroke study in Gothenburg (POSTGOT). J Rehabil Med (2011) 43(4):348–53. 10.2340/16501977-0677 21267528

[13] Simpson LA Miller WC Eng JJ. Effect of stroke on fall rate, location and predictors: a prospective comparison of older adults with and without stroke. PLoS One (2011) 6(4):e19431. 10.1371/journal.pone.0019431 21559367 PMC3084849

[14] Jalayondeja C Sullivan PE Pichaiyongwongdee S. Six-month prospective study of fall risk factors identification in patients post-stroke. Geriatr Gerontol Int (2014) 14(4):778–85. 10.1111/ggi.12164 24666687

[15] Pinto EB Nascimento C Marinho C Oliveira I Monteiro M Castro M Risk factors associated with falls in adult patients after stroke living in the community: baseline data from a stroke cohort in Brazil. Top Stroke Rehabil (2014) 21(3):220–7. 10.1310/tsr2103-220 24985389

[16] Cho K Yu J Rhee H. Risk factors related to falling in stroke patients: a cross-sectional study. J Phys Ther Sci (2015) 27(6):1751–3. 10.1589/jpts.27.1751 26180313 PMC4499976

[17] Barlas RS Honney K Loke YK McCall SJ Bettencourt-Silva JH Clark AB Impact of hemoglobin levels and anemia on mortality in acute stroke: analysis of UK regional registry data, systematic review, and meta-analysis. J Am Heart Assoc (2016) 5(8):e003019. 10.1161/JAHA.115.003019 27534421 PMC5015269

[18] Yuan ZC Mo H Guan J He JL Wu ZJ. Risk of hip fracture following stroke, a meta-analysis of 13 cohort studies. Osteoporos Int (2016) 27(9):2673–9. 10.1007/s00198-016-3603-x 27101998

[19] Kapral MK Fang J Alibhai SM Cram P Cheung AM Casaubon LK Risk of fractures after stroke: results from the Ontario stroke registry. Neurology (2017) 88(1):57–64. 10.1212/WNL.0000000000003457 27881629 PMC5200858

[20] O’Neal WT Qureshi WT Judd SE Bowling CB Howard VJ Howard G Effect of falls on frequency of atrial fibrillation and mortality risk (from the REasons for geographic and racial differences in stroke study). Am J Cardiol (2015) 116(8):1213–8. 10.1016/j.amjcard.2015.07.036 26279105 PMC4589487

[21] Wong CX Gan SW Lee SW Gallagher C Kinnear NJ Lau DH Atrial fibrillation and risk of hip fracture: a population-based analysis of 113,600 individuals. Int J Cardiol (2017) 243:229–32. 10.1016/j.ijcard.2017.05.012 28528985

[22] Cook WL Tomlinson G Donaldson M Markowitz SN Naglie G Sobolev B Falls and fall-related injuries in older dialysis patients. Clin J Am Soc Nephrol (2006) 1(6):1197–204. 10.2215/CJN.01650506 17699348

[23] Farragher J Chiu E Ulutas O Tomlinson G Cook WL Jassal SV. Accidental falls and risk of mortality among older adults on chronic peritoneal dialysis. Clin J Am Soc Nephrol (2014) 9(7):1248–53. 10.2215/CJN.11001013 24763867 PMC4078966

[24] Avin KG Moorthi RN Bone is not alone: the effects of skeletal muscle dysfunction in chronic kidney disease. Curr Osteoporos Rep (2015) 13(3):173–9. 10.1007/s11914-015-0261-4 25691218 PMC4860015

[25] Scherbakov N von Haehling S Anker SD Dirnagl U Doehner W. Stroke induced sarcopenia: muscle wasting and disability after stroke. Int J Cardiol (2013) 170(2):89–94. 10.1016/j.ijcard.2013.10.031 24231058

[26] Toyoda K Ninomiya T Stroke and cerebrovascular diseases in patients with chronic kidney disease. Lancet Neurol (2014) 13(8):823–33. 10.1016/S1474-4422(14)70026-2 25030514

[27] Myint PK Poole KE Warburton EA. Hip fractures after stroke and their prevention. QJM (2007) 100:539–45. 10.1093/qjmed/hcm067 17693418

[28] Schnitzer S Deutschbein J Nolte CH Kohler M Kuhlmey A Schenk L. How does sex affect the care dependency risk one year after stroke? a study based on claims data from a German health insurance fund. Top Stroke Rehabil (2017) 24(6):415–21. 10.1080/10749357.2017.1305645 28330419

[29] Scott D Blizzard L Fell J Jones G. Statin therapy, muscle function and falls risk in community-dwelling older adults. QJM (2009) 102(9):625–33. 10.1093/qjmed/hcp093 19633029

[30] Guise TA. Bone loss and fracture risk associated with cancer therapy. Oncologist (2006) 11(10):1121–31. 10.1634/theoncologist.11-10-1121 17110632

[31] Drake MT. Osteoporosis and cancer. Curr Osteoporos Rep (2013) 11(3):163–70. 10.1007/s11914-013-0154-3 23877475 PMC3783531

[32] Winters-Stone KM Horak F Jacobs PG Trubowitz P Dieckmann NF Stoyles S Falls, functioning, and disability among women with persistent symptoms of chemotherapy-induced peripheral neuropathy. J Clin Oncol (2017) 35(23):2604–12. 10.1200/JCO.2016.71.3552 28586243 PMC5549452

[33] Wu FJ Sheu SY Lin HC Chung SD. Increased fall risk in patients receiving androgen deprivation therapy for prostate cancer. Urology (2016) 95:145–50. 10.1016/j.urology.2016.05.058 27318262

[34] Winters-Stone KM Moe E Graff JN Dieckmann NF Stoyles S Borsch C Falls and frailty in prostate cancer survivors: current, past, and never users of androgen deprivation therapy. J Am Geriatr Soc (2017) 65(7):1414–9. 10.1111/jgs.14795 28263373 PMC5507739

[35] Quinn TJ Taylor-Rowan M Coyte A Clark AB Musgrave SD Metcalf AK Pre-stroke modified Rankin scale: evaluation of validity, prognostic accuracy, and association with treatment. Front Neurol (2017) 13(8):275. 10.3389/fneur.2017.00275 28659859 PMC5468801

[36] Gensicke H Strbian D Zinkstok SM Scheitz JF Bill O Hametner C Intravenous thrombolysis in patients dependent on the daily help of others before stroke. Stroke (2016) 47(2):450–6. 10.1161/STROKEAHA.115.011674 26797662

[37] Sabayan B Oleksik AM Maier AB van Buchem MA Poortvliet RK de Ruijter W High blood pressure and resilience to physical and cognitive decline in the oldest old: the Leiden 85-plus study. J Am Geriatr Soc (2012) 60(11):2014–9. 10.1111/j.1532-5415.2012.04203.x 23126669

[38] Klein D Nagel G Kleiner A Ulmer H Rehberger B Concin H Blood pressure and falls in community-dwelling people aged 60 years and older in the VHM&PP cohort. BMC Geriatr (2013) 13:50. 10.1186/1471-2318-13-50 23692779 PMC3663706

[39] Gasecki D Kwarciany M Nyka W Narkiewicz K. Hypertension, brain damage and cognitive decline. Curr Hypertens Rep (2013) 15(6):547–58. 10.1007/s11906-013-0398-4 24146223 PMC3838597

[40] Gillespie LD Robertson MC Gillespie WJ Lamb SE Gates S Cumming RG Interventions for preventing falls in older people living in the community. Cochrane Database Syst Rev (2009) (2):CD007146 10.1002/14651858.CD007146.pub2 19370674

