# Supplementary material for: Long-Term Factors Associated With Falls and Fractures Poststroke
Source: Front Neurol. 2018 Apr 3;9:210. doi: 10.3389/fneur.2018.00210 (PMC5891595; doi:10.3389/fneur.2018.00210)

Supplementary Material

Long-term factors associated with falls and fractures post-stroke

Emma J. Foster[1], Raphae S. Barlas[1] MPH, Joao H. Bettencourt-Silva[2,3] PhD, Allan B. Clark[3] PhD, Anthony K. Metcalf [2,3] MBChB, Kristian M. Bowles[2,3] PhD, John F. Potter[2,3] DM, Phyo K. Myint[1,3] MD*.

[1]Ageing Clinical & Experimental Research (ACER) Team, Institute of Applied Health Sciences, School of Medicine, Medical Sciences & Nutrition, University of Aberdeen, Aberdeen, UK

[2]Norfolk and Norwich University Hospital, Norwich, UK

[3]Norwich Cardiovascular Research Group, Norwich Medical School, University of East Anglia, Norwich Research Park, Norwich, UK

***Correspondence to:**
Phyo Kyaw Myint,
Room 4:013 Polwarth Building,
School of Medicine, Medical Sciences & Nutrition,
University of Aberdeen, Foresterhill,
Aberdeen, AB25 2ZD, UK
Tel: +44 (0) 1224 437841
Fax: +44 (0) 1224 437911
Mail to: phyo.myint@abdn.ac.uk

Supplementary Fig. 1 – Displaying the process of data exclusion

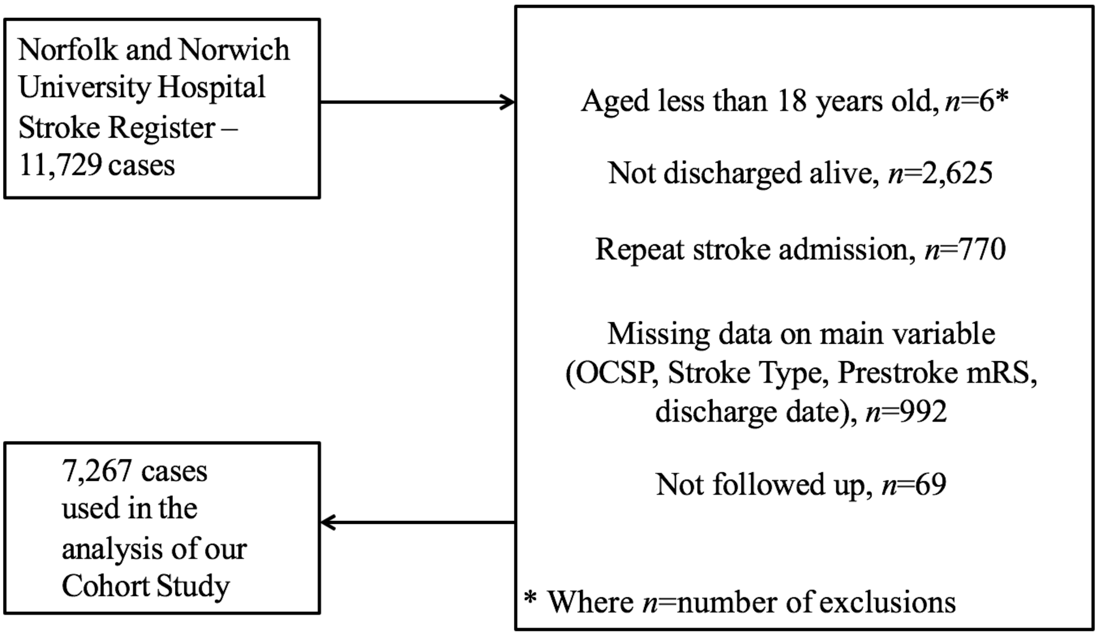

Supplement: Supplementary file 1 [file image_1.PDF]
